# Flexible Thermal Sensitivity of Mitochondrial Oxygen Consumption and Substrate Oxidation in Flying Insect Species

**DOI:** 10.3389/fphys.2022.897174

**Published:** 2022-04-25

**Authors:** Hichem A. Menail, Simon B. Cormier, Mariem Ben Youssef, Lisa Bjerregaard Jørgensen, Jess L. Vickruck, Pier Morin, Luc H. Boudreau, Nicolas Pichaud

**Affiliations:** ^1^ New Brunswick Centre for Precision Medicine, Moncton, NB, Canada; ^2^ Department of Chemistry and Biochemistry, Université de Moncton, Moncton, NB, Canada; ^3^ Zoophysiology, Department of Biology, Aarhus University, Aarhus, Denmark; ^4^ Fredericton Research and Development Centre, Agriculture and Agri-Food Canada, Fredericton, NB, Canada

**Keywords:** temperature, *Apis mellifera carnica*, *Drosophila melanogaster*, *Leptinotarsa decemlineata*, metabolic adaptation, succinate, glycerol-3-phosphate, proline

## Abstract

Mitochondria have been suggested to be paramount for temperature adaptation in insects. Considering the large range of environments colonized by this taxon, we hypothesized that species surviving large temperature changes would be those with the most flexible mitochondria. We thus investigated the responses of mitochondrial oxidative phosphorylation (OXPHOS) to temperature in three flying insects: the honeybee (*Apis mellifera carnica*), the fruit fly (*Drosophila melanogaster*) and the Colorado potato beetle (*Leptinotarsa decemlineata*). Specifically, we measured oxygen consumption in permeabilized flight muscles of these species at 6, 12, 18, 24, 30, 36, 42 and 45°C, sequentially using complex I substrates, proline, succinate, and glycerol-3-phosphate (G3P). Complex I respiration rates (CI-OXPHOS) were very sensitive to temperature in honeybees and fruit flies with high oxygen consumption at mid-range temperatures but a sharp decline at high temperatures. Proline oxidation triggers a major increase in respiration only in potato beetles, following the same pattern as CI-OXPHOS for honeybees and fruit flies. Moreover, both succinate and G3P oxidation allowed an important increase in respiration at high temperatures in honeybees and fruit flies (and to a lesser extent in potato beetles). However, when reaching 45°C, this G3P-induced respiration rate dropped dramatically in fruit flies. These results demonstrate that mitochondrial functions are more resilient to high temperatures in honeybees compared to fruit flies. They also indicate an important but species-specific mitochondrial flexibility for substrate oxidation to sustain high oxygen consumption levels at high temperatures and suggest previously unknown adaptive mechanisms of flying insects’ mitochondria to temperature.

## 1 Introduction

Insects are ectotherms that usually lack the thermogenic ability required to regulate their body temperature (but see Loli and Bicudo, 2005), which may be seen as a physiological barrier to their geographic distribution. Thus, temperature is considered the most important abiotic factor influencing their survival (Bale et al., 2002). However, they have developed adaptive strategies specific to their environment which have allowed them to colonise almost all ecosystems and thermal niches, and be the most abundant taxonomic class of the animal kingdom. These strategies encompass all organisational levels, from physiological to molecular changes as well as fine-tuned adjustments at the cellular level.

Most insects are chill susceptible. Exposure to low temperatures may inhibit ion transport through membranes that dissipates membrane potential and thus leads to neuromuscular failure and chill coma. Ultimately, this can cause a loss of ion and water homeostasis at the cellular level that can induce chill death [reviewed in (Overgaard and Macmillan, 2017)]. However, in temperate climates, some insects can survive chill temperatures through physiological adaptations like freeze resistance (freeze avoidance) and freeze tolerance (Duman et al., 1998), and biochemical and molecular adaptations that include increasing polyunsaturated fatty acid proportion in membrane phospholipids (Hochachka and Somero, 2002) as well as increasing heat shock protein synthesis. These mechanisms take place mainly during overwintering where insects typically undergo a genetically programmed, seasonally synchronised metabolic arrest called diapause (dormant phenotype) that enhances cold stress tolerance/resistance (Denlinger, 2002). However, these insects are not necessarily more resilient to cold spells in summer (active phenotype) mainly because the cold stress tolerance/resistance mechanisms have been lifted (Clark and Worland, 2008). For these unpredictable thermal fluctuations, the process of rapid cold hardening that triggers a quick release of cryoprotectants such as amino acids (i.e., proline, alanine and glutamine), polyols (i.e., glycerol and sorbitol), and sugars (i.e., glucose and trehalose) as well as increased membrane fluidity and modulation of several signalling pathways, is essential to maintain homeostasis and ensure survival of organisms (Lee et al., 1987; Colinet et al., 2007; Lalouette et al., 2007; Michaud and Denlinger, 2007; Overgaard et al., 2007; Clark and Worland, 2008; Teets and Denlinger, 2013).

At high temperatures, the aerobic metabolism is a primary target of thermal effects on ectotherms and is considered a major node for biochemical and metabolic adaptations due to the increased metabolic demand associated with elevated temperatures (Blier et al., 2014; Schulte, 2015; Chung and Schulte, 2020). At the cellular level, this implies that mitochondria have to adjust their oxygen consumption to supply the amount of ATP required to match this increased metabolic demand and restore homeostasis when temperature increases. Therefore, mitochondrial thermal sensitivity has been put forward to tentatively explain organismal failure at high temperatures and is believed to be paramount for thermal adaptation of several organisms (Fangue et al., 2009; Iftikar et al., 2014; Chung et al., 2018; Harada et al., 2019; Pichaud et al., 2019; Hraoui et al., 2020; Hraoui et al., 2021). However, the study of insects has often been overlooked mainly due to technical reasons which resulted in a gap of knowledge concerning mitochondrial functions and the implication of mitochondrial adaptation in insects at high temperature [but see (Davison and Bowler, 1971; Kashmerry and Bowler, 1977; Chamberlin, 2004; Pichaud et al., 2010; Pichaud et al., 2011; Pichaud et al., 2012; Pichaud et al., 2013; Jørgensen et al., 2021)]. The population structure and geographic distribution of insects are thus linked to the plasticity of mitochondria and their capacity to buffer daily temperature fluctuations encountered during summer. However, with more frequent weather extremes, mitochondrial plasticity may not keep up the pace, especially when other stresses hinder their optimal functioning such as availability of nutrients and pesticide exposure for example. Considering their ecological and economic importance, it is thus crucial to characterize the effects of temperature constrains on insects’ mitochondrial metabolism to understand whether and how these organisms will adapt and/or survive to a constantly changing environment.

Mitochondria are involved in several important cellular processes in addition to ATP production such as the oxidation of nutrient-derived substrates which leads to the formation of important metabolites, as well as reactive oxygen species (ROS) production and consumption, among others (Blier et al., 2014; Sokolova, 2018; Chung and Schulte, 2020). At elevated temperatures, inactivation and/or denaturation of proteins, membrane re-organization, high ROS production, failure in substrate transport or insufficient oxygen supply may cause mitochondrial dysfunction which will result in mismatched ATP demand and supply and hence in the failure of aerobic metabolism (Hochachka and Somero, 2002; Jena et al., 2013; Blier et al., 2014; Schulte, 2015; Chung and Schulte, 2020; González-Tokman et al., 2020). Although mitochondria have been suggested as a putative site of dysfunction resulting in organismal failure at high temperature, no specific mechanism has been identified so far due to their complexity and their involvement in a wide range of metabolic and signalling processes. In a recent study using a comparative model of six different *Drosophila* species with different thermal tolerance, we showed that heat tolerance (CT_max_) was correlated with failure of mitochondrial oxygen consumption at the level of complex I and increased utilization of alternative oxidative substrates (Jørgensen et al., 2021). Specifically, the mitochondrial oxygen consumption supported by complex I substrates was drastically reduced at temperatures near CT_max_ of the specific species, and glycerol-3-phosphate (G3P) oxidation by the mitochondrial glycerol-3-phosphate dehydrogenase (mtG3PDH) was augmented (Jørgensen et al., 2021). This suggests that mitochondria are extremely flexible in the context of temperature changes and have the ability to switch from a “standard” metabolic pathway to a more heat-tolerant pathway, potentially to sustain higher energetic turnover and maintain homeostasis when temperature increases. Although this mitochondrial flexibility driven by temperature has been demonstrated in different *Drosophila* species (Jørgensen et al., 2021), it is important to use a wider phylogenetic comparison of insect orders to assess the fine regulations of such complex responses and then be able to generalize to a wide range of animals (González-Tokman et al., 2020).

Insects can rely on a large set of substrates to fuel their energetic metabolism. They are either highly specialized in oxidising one particular substrate or can switch from a substrate to another that better suit their energetic demands, season, activity, and environmental conditions (Moyes et al., 1990; McDonald et al., 2018; Sinclair and Marshall, 2018). In temperate climates, most insects mainly rely on carbohydrates to fuel their mitochondria under normal conditions. However, at low temperatures, during diapause or for sustained flight during migration, lipids are preferred (Sacktor, 1955; Beenakkers et al., 1984; Candy et al., 1997; Haunerland, 1997; Sinclair and Marshall, 2018). Other metabolic fuels can also be used by insects. For example, G3P can be used as a substrate that directly donates electrons to the mitochondrial electron transport system (ETS) at the level of mtG3PDH (Sacktor and Cochran, 1958; Soares et al., 2015; Masson et al., 2017; McDonald et al., 2018; Cormier et al., 2021). G3P can be produced from glycolysis *via* the conversion of dihydroxyacetone phosphate, or from di- and tri-glyceride degradation *via* phosphorylation of the residual glycerol skeleton (McDonald et al., 2018). Another example is the amino acid proline that can be used as an anaplerotic donor of electrons to complex I in some species (Sacktor and Childress, 1967; Micheu et al., 2000; Suarez et al., 2005; Soares et al., 2015), or as a direct donor of electron to the proline dehydrogenase (ProDH) in other species (Bursell, 1963; Bursell, 1981; Soares et al., 2015; Teulier et al., 2016; McDonald et al., 2018). Succinate is another oxidative substrate that can act as an electron donor to the ETS *via* complex II, and its importance has also recently been put forward in several organisms during challenging environmental conditions such as hypoxia/anoxia and reoxygenation stresses (Bundgaard et al., 2018; Cox and Gillis, 2020; Martin et al., 2021; Adzigbli et al., 2022). These substrates are thus an important part of the oxidative capacity of mitochondria and must be systematically taken into account when evaluating mitochondrial thermal sensitivity.

In this study, we used three insect species representing different taxonomic orders to evaluate the flexibility of mitochondrial substrate oxidation at different temperatures. Specifically, we measured oxygen consumption of permeabilized flight (thoracic) muscles at eight different temperatures (6, 12, 18, 24, 30, 36, 42, and 45°C) using different oxidative substrates in the following species: the honeybee, *Apis mellifera carnica* (Hymenoptera), an important pollinator which is paramount for ecosystem balance; the fruit fly, *Drosophila melanogaster* (Diptera), which is one of the most commonly used model organisms; and the Colorado potato beetle, *Leptinotarsa decemlineata* (Coleoptera), often studied for its detrimental role as pest. Honeybees are heterotherms that can generate heat by shivering their thoracic muscles whenever temperature goes beneath 30°C which allows them to maintain an in-hive temperature of 32–36°C even during winter (Stabentheiner et al., 2012; Kovac et al., 2014). Fruit flies, however, are very vulnerable to chill and seek shelter to survive cold temperatures (Koštál et al., 2011; Koštál et al., 2012; Strachan et al., 2015). On the contrary, potato beetles are freeze-resistant insects that can go into diapause which enhances their cold stress tolerance by suppressing their metabolism and degrading fibres and mitochondria of their flight muscles (Stegwee et al., 1963; Boiteau and Coleman, 1996; Denlinger, 2002). Here, we assessed the mitochondrial capacities of these species during summer (active phenotype) where experimental thermal stresses (acclimated to a common temperature) reflect the phenotypic plasticity of insects during rapid hardening. During summer, these three species also have different metabolic demands especially considering flight, which is an extremely energy-demanding activity mostly powered by aerobic metabolism (Harrison and Roberts, 2000). As a result, flying insects are known to have the most rapidly contracting muscles in nature with one of the highest mitochondrial volume densities (Beenakkers et al., 1984; Suarez et al., 1996; Candy et al., 1997; Suarez, 2000; Suarez et al., 2000). Considering 1) the metabolic capacities and life history trait differences between these species, 2) energy source variations between insect species (Moyes et al., 1990), and 3) previous results on mitochondrial thermal sensitivity and metabolic flexibility in different *Drosophila* species (Jørgensen et al., 2021), we hypothesized that the complexes of the ETS accepting electrons from oxidative substrates (complex I, ProDH, complex II, mtG3PDH): 1) will contribute differently to mitochondrial respiration; 2) will have different thermal sensitivities; and 3) that mitochondrial flexibility to use a specific substrate as metabolic fuel in response to thermal changes will be specific to each species.

## 2 Material and Methods

### 2.1 Animal Maintenance and Collection

Three insect species were used in this study; the honeybee *Apis mellifera carnica*; the fruit fly *Drosophila melanogaster*, and the potato beetle *Leptinotarsa decemlineata*. All species were collected during summer, thus representing an active phenotype (as opposed to a winter dormant phenotype) and were maintained in laboratory incubators at 24°C prior to experiments between spring and autumn 2020.

#### 2.1.1 *Apis mellifera carnica*

Honeybees from the carniolian subspecies *Apis mellifera carnica* were maintained in experimental hives (Amohive®, ON, Canada) at the Université de Moncton (Moncton, NB, Canada). A sealed brood frame was taken to the laboratory at the end of summer and incubated at 30°C. Newly emerged honeybees were put in experimental plastic cages in groups of 30 and were maintained at 30°C for 2 days. They were then transferred to 24°C and 50% relative humidity until they were sampled for mitochondrial respiration experiments at 25 days old. During all the experiments, they were fed *ad libitum* with sucrose syrup (50% w/v) and pollen patties consisting of dried pollen granules mixed with sucrose syrup. Food was changed weekly, and cages were replaced when needed. Honeybees were always maintained in the dark.

#### 2.1.2 *Drosophila melanogaster*

Fruit flies (wild-type w^1118^, Bloomington *Drosophila* Stock Center, Bloomington, IN, United States) were maintained in plastic vials containing a standard cornmeal medium consisting of 5 gL^−1^ agar,6 gL^−1^sugar, 27 gL^−1^yeast, 53 gL^−1^cornmeal in 1 L of tap water and supplemented with methyl-p-hydroxybenzoate dissolved in 95% ethanol (10% w/v) and propionic acid (0.4% v/v). Male flies were maintained at 24°C, 50% relative humidity, photoperiod 12 h light:12 h dark, and constant density (30 flies per vial) until they were sampled for experiments at 11 days old.

#### 2.1.3 *Leptinotarsa decemlineata*

Potato beetles were provided by the nursery of the Fredericton Research and Development Centre (FRDC, Fredericton, NB, Canada). Upon reception, they were maintained at 24°C, 50% relative humidity, photoperiod 12 h light:12 h dark, and fed potato plants for at least 1 week before individuals of unknown age and sex were sampled for the experiments.

### 2.2 Determination of Mitochondrial Oxygen Consumption in Permeabilized Thoraces

Mitochondrial oxygen consumption was measured using protocols previously developed for *Drosophila* (Simard et al., 2018; Cormier et al., 2019) and adapted for honeybees and beetles.

#### 2.2.1 Tissue Preparation

Tissues were sampled from freshly sacrificed insects and all manipulations were performed on ice. For honeybees and potato beetles, thoraces were dissected, and a sample of muscle fibres was taken for experiments (*n* = 9–10 and 8–10 for each temperature, respectively) while for fruit flies, three thoraces were pooled for each sample (*n* = 5–10 for each temperature). The sampled tissues were placed in a Petri dish containing a BIOPS solution [2.77 mM CaK_2_-EGTA, 7.23 mM K_2_-EGTA, 5.77 mM Na_2_-ATP, 6.56 mM MgCl_2_, 20 mM taurine, 15 mM Na_2_-phosphocreatine, 20 mM imidazole, 0.5 mM dithiothreitol, and 50 mM K-MES, pH 7.1 (Pesta and Gnaiger, 2012)]. We then performed a mechanical permeabilization of tissues using fine forceps followed by an incubation at 4°C for 15 min on an orbital shaker in BIOPS supplemented with saponin (62.5 μg ml^−1^) for chemical permeabilization. Tissues were then transferred to respiration medium [120 mM KCl, 5 mM KH_2_PO_4_, 3 mM HEPES, 1 mM MgCl_2_, and 0.2% BSA (w/v), pH 7.2] and shaken for an additional 5 min to wash out saponin. Finally, we gently dried and weighed the tissues using a semi-micro balance (Secura 225D-1, Sartorius, Göttingen, Germany) before transferring the fibres to the chambers of a high-resolution respirometer.

#### 2.2.2 Measurements of Mitochondrial Respiration Rates

Respiration rates were measured at eight different temperatures (6, 12, 18, 24, 30, 36, 42, and 45°C) using Oxygraph-O2K high-resolution respirometers (Oroboros Instruments, Innsbruck, Austria). This range of temperatures includes the acclimation temperature (24°C) and is representative of both low to high temperatures experienced by these insects in their environment, as well as critically high temperatures. The respirometers were calibrated with air-saturated respiration medium at each temperature tested. Complex I (NAD-linked) substrates i.e., pyruvate (10 mM) and malate (2 mM) were added into the chambers before the permeabilized tissues of fruit flies and beetles were transferred. In addition to pyruvate and malate, glutamate (10 mM) was also added for honeybees (Syromyatnikov et al., 2019). The signal was allowed to stabilize and the resulting respiration rate corresponding to the non-phosphorylating respiration (or LEAK) at the level of complex I (CI-LEAK) was measured.

We then added ADP (5 mM) to measure oxidative phosphorylation respiration rates (OXPHOS) sustained by complex I substrates (CI-OXPHOS). Addition of cytochrome c (20 µM) was then performed to verify the integrity of the outer mitochondrial membrane (CIc-OXPHOS): if this injection stimulated a ≥15% increase in oxygen consumption, the sample was discarded (Kuznetsov et al., 2008). We then sequentially injected substrates allowing the entry of electrons into the ETS at several complexes during OXPHOS: proline (10 mM) to fuel the ProDH (CI + ProDH-OXPHOS); succinate (5 mM) to fuel complex II (CI + ProDH + CII-OXPHOS); and G3P (15 mM) to fuel the mtG3PDH (CI + ProDH + CII + mtG3PDH-OXPHOS).

Following this, we added the uncoupler carbonyl cyanide 4-(trifluoromethoxy) phenylhydrazone (FCCP, steps of 0.5 µM) to achieve the maximal ETS respiration rate (Max-ETS). Rotenone (0.5 µM), malonate (5 mM), and antimycin A (5 µM) were then injected to inhibit complexes I, II, and III, respectively and measure the residual oxygen consumption rate, which was subtracted from the other rates. Finally, ascorbate (2 mM) and N,N,N',N,-Tetramethyl-p-phenylenediamine (TMPD) (0.5 mM) were added to measure the maximal capacity of complex IV which was corrected for TMPD auto-oxidation after inhibition of complex IV by sodium azide (50 mM).

### 2.3 Analysis of Respiration Rates and Mitochondrial Ratios

After mitochondrial respiration experiments were performed, oxygen consumption rates (OCRs) were estimated by selecting the range of measured O_2_ consumption rates that was the most stable after the addition of each substrate (as mentioned above). All OCRs are presented as means of specific respiration rates expressed as pmol O_2_.s^−1^.mg^−1^ of tissue ± s.e.m. When assessing respiration levels for beetles, some OCRs did not stabilize, as already observed in some *Drosophila* species (Jørgensen et al., 2021), notably after proline addition at 30 and 42°C. Specifically, mitochondrial O_2_ consumption increased significantly and immediately, but steadily decreased thereafter, not allowing the estimation of a reliable respiration rate. In this case, the respiration rate was taken 10 min after the start of the O_2_ consumption decrease and another one was taken just prior to the addition of the next substrate, succinate. However, only the second rate was used for further calculations and comparisons. The OCRs obtained allowed the calculation of several mitochondrial ratios, the contribution of different substrates to O_2_ consumption, the effect of temperature on each of these values, and comparison between species.

#### 2.3.1 Oxidative Phosphorylation Coupling Efficiency

First, the OXPHOS coupling efficiency at the level of complex I was calculated as:

$$CI\;coupling\;efficiency=1-\frac{CI­LEAK}{CI­OXPHOS}$$

If this ratio is close to 1.0, it indicates a tight coupling between the electron transport from complex I and the phosphorylation process. However, a decrease of this ratio might indicate a loss of coupling and dysfunction of complex I.

#### 2.3.2 Substrate Contribution Ratio

To be able to estimate each substrate contribution (proline, succinate, and G3P) to mitochondrial respiration, the substrate contribution ratio (SCR) was calculated using the following formula:

$$SCR=\frac{OCR2-OCR1}{OCR1}$$

where OCR1 represents the oxygen consumption rate prior to the injection of the substrate and OCR2 corresponds to the oxygen consumption rate after the injection of the same substrate.

If SCR = 1.0, the oxygen consumption rate increased by 100% (oxygen consumption doubled after the injection of the corresponding substrate).

#### 2.3.3 Uncoupling Control Ratio

The uncoupling control ratio (UCR) was calculated as:

$$UCR=\frac{Max­ETS}{CI+ProDH+CII+mtG3PDH­OXPHOS}$$

An uncoupled ratio higher than 1.0 reflects a limitation of complex V to process the proton gradient, while a ratio close to 1.0 indicates that the substrates provided are stimulating maximal electron transport by the ETS complexes. In all cases, the ETS maximal capacity was reached with the substrates provided as suggested by UCRs varying between 0.85 and 1.5 for all three species, except at low temperatures (6 and 12°C) in beetles (results not shown).

#### 2.3.4 Interspecies Comparisons

To accurately compare OCRs between the three species, each OCR (after the addition of each substrate) was divided by the OCR corresponding to the maximal activity of CIV, i.e., using CIV maximal oxygen consumption rate as an internal normalizer. Complex IV is known to display excess capacity compared to the other complexes of the ETS (Gnaiger et al., 1998), which makes it a good normalizer to evaluate specific changes of the different complexes during temperature changes.

### 2.4 Statistical Analyses

Statistical analyses were performed using GraphPadPrism 8.0.2 for Windows (GraphPad software, La Jolla California, United States, www.Graphpad.com). Oxygen consumption rates were compared; 1) between temperatures for each substrate (for each species); 2) between species for each temperature and for each substrate (between species comparison); and 3) between temperatures for each substrate contribution using one-way ANOVAs followed by a HSD (Honest Significant Difference) Tukey’s test. Normality of residuals and homogeneity of variances were verified, and data were transformed when required. If these conditions were not met, a non-parametric test (Kruskall-Wallis) followed by a Dunn’s test were performed.

## 3 Results

### 3.1 Thermal Sensitivity of Mitochondrial Oxygen Consumption for Each Species

#### 3.1.1 *Apis mellifera carnica*

**FIGURE 1:**
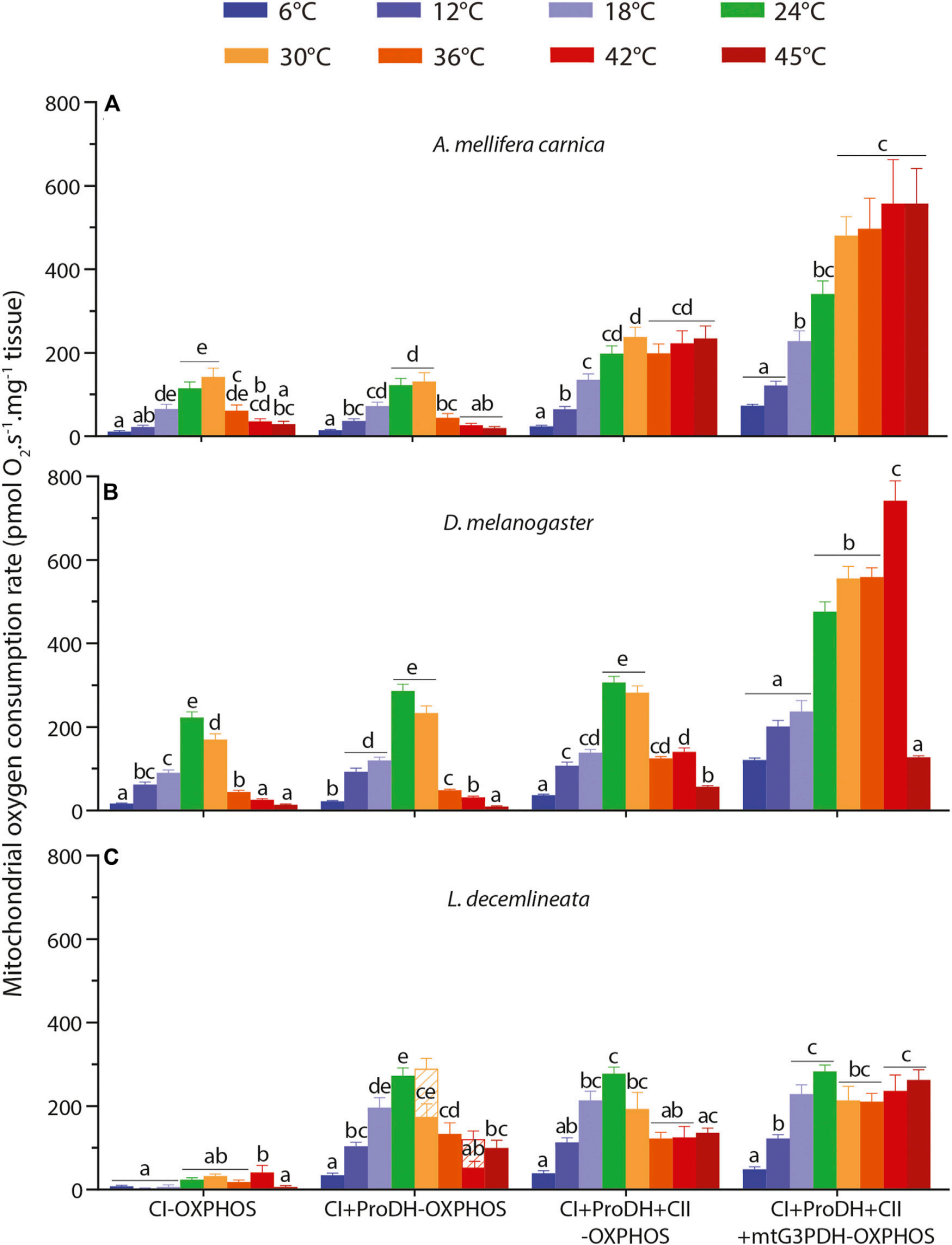
Thermal sensitivity of mitochondrial oxygen consumption rate (OCR) measured from 6 to 45°C in permeabilized thoraces of the three insect species. **(A)** *A. mellifera carnica* (*n* = 7–10), **(B)** *D. melanogaster* (*n* = 5–10) and **(C)** *L. decemlineata* (*n* = 5–10). OCRs were measured with pyruvate + malate (+glutamate in honeybees) +ADP (CI-OXPHOS), +proline (CI + ProDH-OXPHOS), +succinate (CI + ProDH + CII-OXPHOS), +G3P (CI + ProDH + CII + mtG3PDH-OXPHOS). OCRs are reported as mean ± s.e.m. and were compared within each species between assay temperatures using a one-way ANOVA followed by a HSD (Honest Significant Difference) Tukey’s test. Dissimilar letters indicate significant differences between OCRs. For CI + ProDH + CII-OXPHOS in beetles, one-way ANOVA conditions were not met, and a non-parametric test (Kruskall-Wallis) followed by a Dunn’s test were performed. When assessing respiration levels for *L. decemlineata*, OCRs at 30 and 42°C did not stabilize after proline addition (CI + ProDH-OXPHOS), not allowing the estimation of a reliable respiration rate. In this case, the respiration rate was taken 10 min after the start of the O_2_ consumption decrease (represented with hatched bars) and another one was taken just prior to the addition of the next substrate, succinate. In this case, only the second rate was used for the calculations and the comparisons.

In honeybees, all respiration rates were influenced by the assay temperature (Supplementary Table S1). CI-OXPHOS and CI + ProDH-OXPHOS displayed the exact same pattern, with a steady increase from 6 to 30°C followed by a sharp decrease from 30 to 45°C (Figure 1A). Specifically, both respiration rates were maximal at 30°C with significant differences compared to 6, 12, 42, and 45°C for CI-OXPHOS and to 6, 12, 36, 42, and 45°C for CI + ProDH-OXPHOS (Figure 1A). Of interest, the addition of proline did not markedly increase the mitochondrial oxygen consumption (Figure 1A).

However, when succinate and G3P were added (CI + ProDH + CII-OXPHOS and CI + ProDH + CII + mtG3PDH-OXPHOS, respectively) a different trend emerged. First, we observed steady significant increases of OCRs from 6 to 30°C for both substrates (Figure 1A). This was followed by a plateau with succinate and a small increase with G3P from 30 to 45°C with no significant differences detected across these temperatures (Figure 1A).

#### 3.1.2 *Drosophila melanogaster*

In fruit flies, the assay temperature also had a profound impact on all respiration rates (Supplementary Table S1). A similar trend than for honeybees was observed for CI-OXPHOS and CI + ProDH-OXPHOS, with a sharp increase in OCRs from 6 to 24°C (especially from 18 to 24°C), followed by a drop at 36, 42, and 45°C (Figure 1B). Both respiration rates were maximal at 24°C without significant differences from 30°C in CI + ProDH-OXPHOS (Figure 1B).

The addition of succinate did not change this pattern (Figure 1B). However, the addition of G3P triggered a profound change in OCR, as respiration rates became maximal at 42°C which was significantly higher than the OCRs measured between 24 and 36°C (Figure 1B). In addition, a sharp drop of OCR was observed at 45°C reaching a value similar to that at the lower temperatures (Figure 1B).

#### 3.1.3 *Leptinotarsa decemlineata*

In potato beetles, respiration rates were influenced by temperature (Supplementary Table S1) but displayed different patterns depending on the substrates. Values for CI-OXPHOS were generally low and reached a maximum at 42°C with significant differences detected at 6, 12, 18, and 45°C (Figure 1C). CI-ProDH-OXPHOS, on the other hand, exhibited a striking and steady increase in respiration rates from 6 to 24–30°C before decreasing at 36, 42, and 45°C (Figure 1C).

The addition of succinate (CI + ProDH + CII-OXPHOS) did not trigger a significant change in OCR. First, we observed a steady increase from 6 to 24°C, followed by a slight decrease from 30 to 45°C with significant differences detected between 24 and 36–42°C (Figure 1C). When G3P was added, we observed relatively high and stable OCRs (CI + ProDH + CII + mtG3PDH-OXPHOS) from 18 to 45°C without any significant differences (Figure 1C).

### 3.2 Temperature Impact on Complex I Coupling Efficiency

**FIGURE 2:**
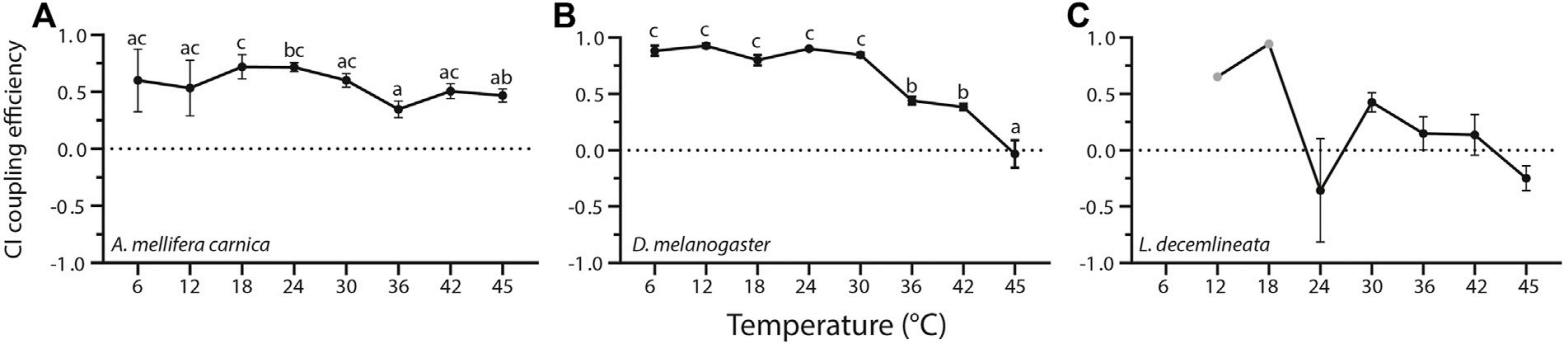
Complex I coupling efficiency calculated from mitochondrial oxygen consumption rates measured in permeabilized thoraces of the three insect species. **(A)** *A. mellifera carnica* (*n* = 3–10), **(B)** *D. melanogaster* (*n* = 3–10), and **(C)** *L. decemlineata* (*n* = 0–10). Complex I coupling efficiency was calculated as 1-(CI-LEAK/CI-OXPHOS), and reported as mean ± s.e.m. Complex I coupling efficiency values were compared within species between assay temperatures using a one-way ANOVA followed by a HSD (Honest Significant Difference) Tukey’s test. Dissimilar letters indicate significant differences within species. In *L.decemlineata*, CI coupling efficiency values were not calculated for 6°C, and only one value was used for 12 and 18°C (grey points).

Temperature influences CI coupling efficiency in a species-specific manner (Figure 2, Supplementary Table S1). In honeybees, CI coupling efficiency was quite stable at all temperatures except at 36°C where it was significantly lower than those measured at 18 and 24°C (Figure 2A). Contrastingly, CI coupling efficiency, although higher than in honeybees between 6 and 30°C, decreased dramatically at high temperatures in fruit flies between 30 and 45°C (Figure 2B). On the other hand, no statistical differences between assay temperatures were detected in beetles, with some negative CI coupling efficiency values calculated at 24 and 45°C (Supplementary Table S1, Figure 2C).

In beetles, negative CI coupling efficiency reflected a decrease in OCR when ADP was added (CI-OXPHOS < CI-LEAK). Moreover, at 6, 12, and 18°C, some OCR values were negative (CI-LEAK and/or CI-OXPHOS). In these cases, these values were not used to calculate the CI coupling efficiency. Thus, only CI coupling efficiency between 24 and 45°C were used for statistical analysis in beetles (Figure 2C, Supplementary Table S1). Overall, this highlights the lack of mitochondrial respiration induced by complex I substrates in this species.

### 3.3 Temperature Impact on Substrate Contribution Ratios

**FIGURE 3:**
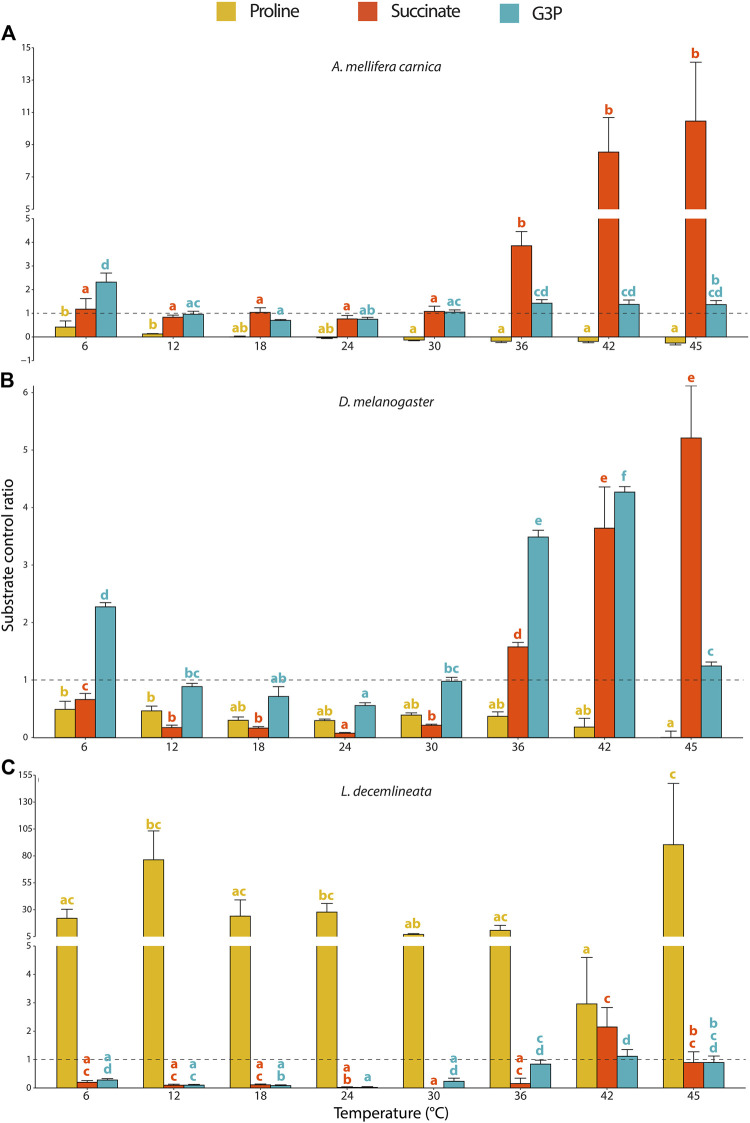
Substrate contribution ratio (SCR) for proline, succinate and G3P calculated from mitochondrial oxygen consumption rates measured in permeabilized thoraces of the three insect species. **(A)** *A. mellifera carnica* (*n* = 8–10), **(B)** *D. melanogaster* (*n* = 5–10), and **(C)** *L. decemlineata* (*n* = 4–10). Substrate contribution ratios were calculated as SCR=(OCR2-OCR1)/OCR1 where OCR1 represents the oxygen consumption rate prior to the injection of substrate *x* and OCR2 corresponds to the oxygen consumption rate after the injection of substrate *x*; SCR = 1.0 is represented with a dashed line and indicates that OCR increased by 100% (doubled) after injection of a specific substrate. Substrate contribution ratios are reported as mean ± s.e.m. and were compared within species for each substrate and between assay temperatures using a one-way ANOVA followed by a HSD (Honest Significant Difference) Tukey’s test. Dissimilar letters indicate significant differences between assay temperatures (yellow for proline, red for succinate and blue for G3P) for each species. In honeybees (proline) and beetles (succinate and G3P), one-way ANOVA conditions were not met, and a non-parametric test (Kruskall-Wallis) followed by a Dunn’s test were performed.

The contributions of each substrate to the OCR were calculated for each species and displayed very different patterns in the three insects but were all influenced by the assay temperature (Figure 3, Supplementary Table S2).

In honeybees, both succinate and G3P highly increased the mitochondrial oxygen consumption and allowed to maintain OCRs at relatively stable values from 24 to 45°C (Figure 1A). Specifically, succinate was the substrate mainly contributing to the increased mitochondrial respiration observed between 36 and 45°C (Figure 3A).

In fruit flies, G3P stimulated the highest increase of OCR contrasting proline and succinate which only slightly increased mitochondrial oxygen consumption (Figure 1B). The substrate contribution ratio for proline did not change across any of the temperatures tested (Figure 3B). For succinate, SCR decreased significantly from 6 to 24°C, but then sharply and steadily increased from 24 to 45°C, with succinate being the greatest contributor to mitochondrial respiration at 45°C (Figure 3B). We observed an identical pattern for G3P contribution (steady decrease from 6 to 24°C and steady increase onwards) except that it sharply dropped at 45°C (Figure 3B), which is in accordance with previous observations in the same species (Jørgensen et al., 2021).

For beetles, proline most prominently increased OCR across all temperatures, but succinate and G3P also allowed a substantial increase of OCR at high temperatures (from 42 to 45°C and from 36 to 45°C, respectively; Figure 1C). However, the pattern for the SCRs in beetles was more erratic (compared to the other species) due to the low OCRs detected with complex I substrates (Figure 1C). Therefore, we observed that proline was by far the main substrate contributing to the OCRs, although few statistical differences were detected between assay temperatures due to disproportionately high calculated SCRs (Figure 3C).

In honeybees and beetles, some SCRs were negative not because OCRs decreased after injection of the corresponding substrate but rather because the OCR prior to substrate injection was negative and thus, these were not used for the calculations.

### 3.4 Thermal Sensitivity of Complex IV Maximal Oxygen Consumption Rate

**FIGURE 4:**
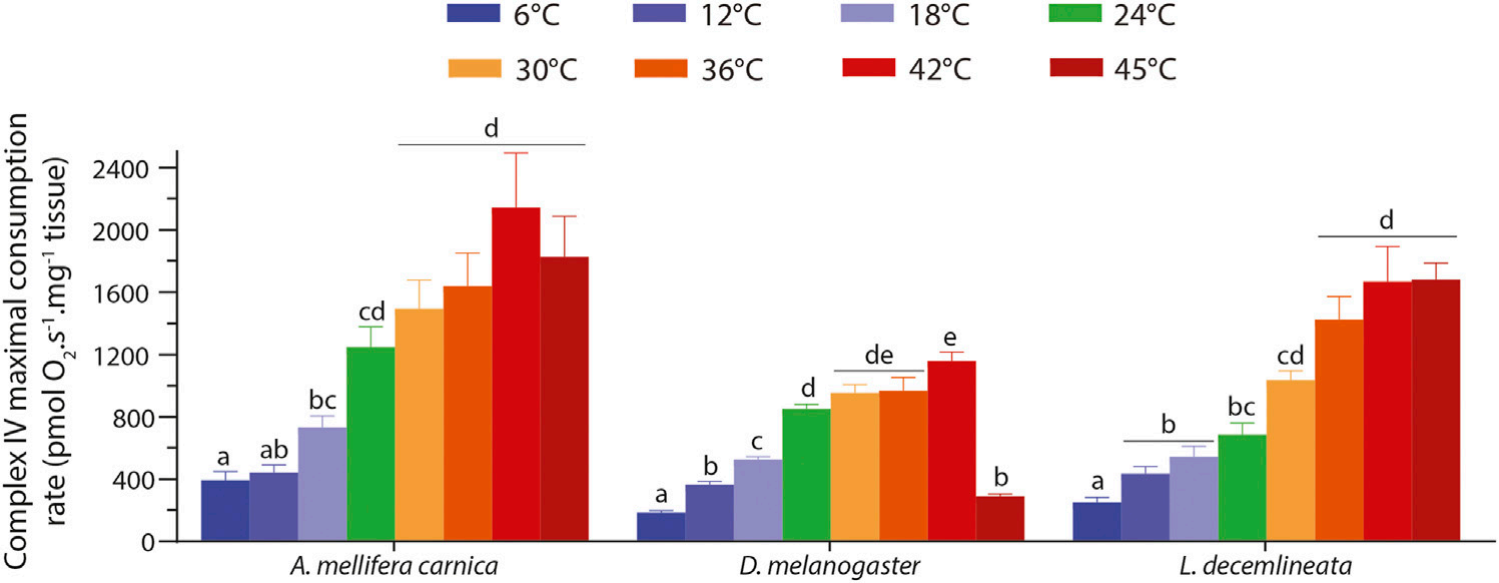
Complex IV maximal oxygen consumption rate measured in permeabilized thoraces of *A. mellifera carnica* (*n* = 9–10), *D. melanogaster* (*n* = 5–10) and *L. decemlineata* (*n* = 8–10). Complex IV maximal oxygen consumption rates were measured after inhibition of complexes I, II, and III and the addition of ascorbate and TMPD. This rate was corrected for TMPD auto-oxidation by further inhibiting complex IV with sodium azide. These rates are reported as mean ± s.e.m and were compared within species between assay temperatures using a one-way ANOVA followed by a HSD (Honest Significant Difference) Tukey’s test. Dissimilar letters indicate significant differences between Complex IV maximal oxygen consumption rates for each species.

Complex IV maximal OCR in the three insects displayed similar patterns for each species, albeit fruit flies differed slightly from the two other insects (Figure 4). Specifically, complex IV maximal OCRs increased steadily from 6 to 42°C in all three species with different levels of significance (Figure 4). At 45°C however, the OCR decreased slightly, albeit not significantly in honeybees, dropped significantly in fruit flies, and plateaued in beetles (Figure 4).

### 3.5 Interspecies Comparisons

**FIGURE 5:**
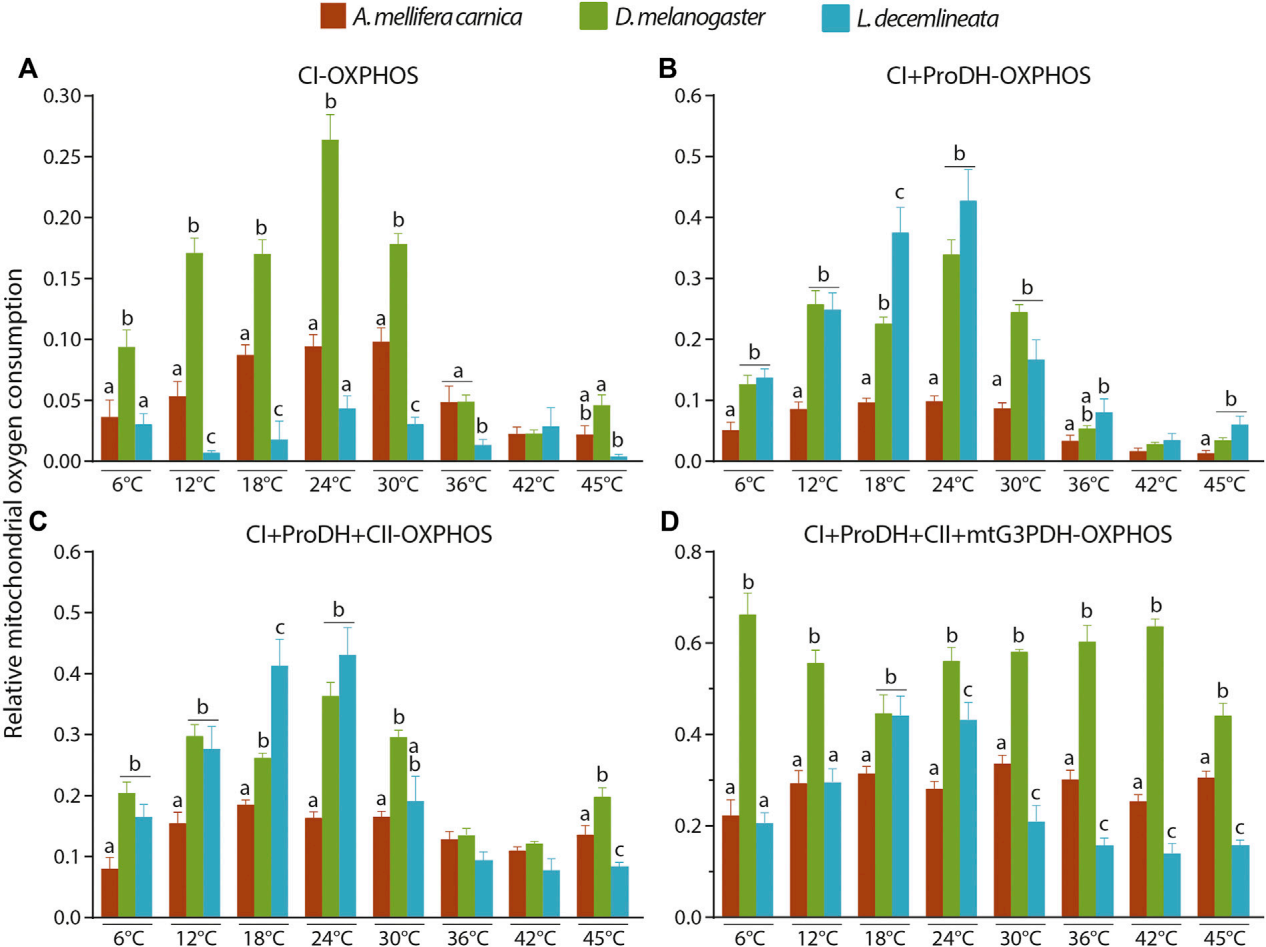
Interspecies comparisons of relative OCRs in the permeabilized thoraces of the three insect species (*A. mellifera carnica*, *D. melanogaster*, and *L. decemlineata*). **(A)** CI-OXPHOS, **(B)** CI + ProDH-OXPHOS, **(C)** CI + ProDH + CII-OXPHOS, and **(D)** CI + ProDH + CII + mtG3PDH-OXPHOS. Relative OCRs are reported as mean ± s.e.m and were obtained by dividing each OCR by the corresponding complex IV maximal OCR for 6 (*n* = 6–10), 12 (*n* = 6–10), 18 (*n* = 5–10), 24 (*n* = 5–10), 30 (*n* = 6–10), 36 (*n* = 6–10), 42 (*n* = 6–10) and 45°C (*n* = 5–9). Relative OCRs were compared between species for each assay temperature using a one-way ANOVA followed by a HSD (Honest Significant Difference) Tukey’s test. Dissimilar letters indicate significant differences between species at each assay temperature. For CI + ProDH + CII-OXPHOS at 30°C, one-way ANOVA conditions were not met, and a non-parametric test (Kruskall-Wallis) followed by a Dunn’s test were performed.

The complex IV maximal OCR was also used as an internal normalizer to properly compare the mitochondrial capacity between species (Figure 5, Supplementary Table S3). When comparing the relative CI-OXPHOS between the three insect species, fruit flies displayed significantly higher relative OCR values from 6 to 30°C, which then decreased to values similar to honeybees from 36 to 45°C (Figure 5A). Moreover, beetles generally had the lowest relative CI-OXPHOS OCRs at all temperatures (Figure 5A).

When proline was added (CI + ProDH-OXPHOS), the relative OCRs greatly increased for beetles reaching levels similar to those of fruit flies (at all temperatures except 18°C where it was statistically higher), and honeybees had the lowest relative CI-ProDH-OXPHOS OCRs at all temperatures (Figure 5B).

When succinate was added, relative CI + ProDH + CII-OXPHOS followed pattern similar to that for proline with fruit flies and beetles displaying the highest values from 6 to 30°C (Figure 5C). At higher temperatures, relative OCRs for the three species were equivalent, except at 45°C where fruit flies had statistically higher relative OCR, followed by honeybees, and beetles displaying statistically lower relative OCR (Figure 5C).

Finally, when G3P was added, relative CI + ProDH + CII + mtG3PDH-OXPHOS was statistically higher for fruit flies at all temperature tested (except 18°C where it was similar to beetles; Figure 5D). Interestingly, when comparing relative CI + ProDH + CII + G3PDH-OXPHOS between beetles and honeybees, a clear pattern was detected in which relative OCRs were higher at 18 and 24°C for beetles, but lower from 30°C upwards (Figure 5D).

## 4 Discussion

This study assessed the thermal sensitivity of mitochondrial respiration and the flexibility of mitochondrial substrate oxidation in the honeybee (*A. mellifera carnica)*, the fruit fly (*D. melanogaster*) and the potato beetle (*L. decemlineata*) flight muscle by measuring mitochondrial oxygen consumption at eight temperatures from 6 to 45°C. Overall, our results show that temperature has an acute impact on mitochondrial respiration in all three insect species (albeit to a different extent), and that mitochondria can rely on several different metabolic pathways to sustain high activity at elevated temperatures, with important changes in the oxidation of substrates which were specific to each species.

### 4.1 Complex I-Oxidative Phosphorylation Breakdown at High Temperature

In honeybees, CI-substrates sustained high respiration rates between 18 and 30°C, which then significantly decreased at 42 and 45°C. In fruit flies, CI-OXPHOS followed the same pattern but declined at a lower temperature than honeybees (≥36°C). Contrastingly, potato beetles had a very low CI-OXPHOS rate which was somehow insensitive to temperature increases (Figure 1). This indicates that CI-OXPHOS is sensitive to temperature in honeybees and fruit flies and cannot sustain mitochondrial oxygen consumption at high temperatures. This is consistent with previous studies on different insects such as *Drosophila spp.* (Jørgensen et al., 2021) and blowfly (El-Wadawi and Bowler, 1995) as well as on other ectotherms such as fishes and crustaceans (Iftikar et al., 2010; Martinez et al., 2016; Chung et al., 2017). Jørgensen et al. (2021) further investigated the cause of this failure in CI-OXPHOS in *Drosophila* and demonstrated that CI catalytic activity surprisingly increased until 45°C (the highest temperature measured) which rejected hyperthermic denaturation of complex I as a potential cause. They instead suggested that TCA cycle enzymes providing NADH to CI might be involved in this failure, but this potential limitation has been challenged in other ectotherm species (Ekström et al., 2017; Hraoui et al., 2020) which keeps the question open whether CI-OXPHOS breakdown would be multifactorial (Jørgensen et al., 2021). For example, the decreased CI coupling efficiency from 36°C in fruit flies (Figure 2B) substantiated by decreased CI-OXPHOS (Figure 1B) and relatively stable CI-LEAK (Supplementary Figure S1) might suggest a potential problem at the level of the substrate transport and/or integrity of the mitochondrial membranes (Chung et al., 2018; Chung and Schulte, 2020).

In honeybees however, it is surprising that such a decline of CI-OXPHOS was detected from 36°C (Fig. 1A), as the flight muscles of this species have been shown to function at ∼35°C during flight (Stabentheiner et al., 2003; Stabentheiner et al., 2010). Moreover, the CI-OXPHOS decline was associated with a relatively similar pattern in CI-LEAK (Supplementary Figure S1), which translated to stable CI coupling efficiency, even at high temperatures (Figure 2). This suggests that a metabolic switch rather than a CI dysfunction occurs in honeybees and indicates that at flight temperature, honeybees favour the oxidation of other substrates such as succinate and G3P instead of CI-substrates.

Both CI-OXPHOS and CI-LEAK (Supplementary Figure S1) in beetles were very low and it is difficult to detect a clear trend about the effect of temperature for these OCRs. However, it has already been shown that this species has low rates of oxygen consumption when oxidizing pyruvate and malate but rather rely on proline as oxidative substrate for flight (Weeda et al., 1980).

### 4.2 Proline Dehydrogenase Offsets Lack of Oxygen Consumption With CI Substrates in Potato Beetles

In honeybees and fruit flies, proline barely increased OCR and generally did not change respiration patterns observed with CI-substrates at any temperatures tested (Figures 1, 3), as previously shown by Jørgensen et al. (2021) in *Drosphila spp*. Moreover, our results are congruent with Teulier et al. (2016) who demonstrated that honeybees are incapable of oxidizing proline to fuel mitochondrial respiration. Contrastingly, in potato beetles, we observed a large increase in OCR following proline injection especially at mid-range temperatures (Figure 1C), albeit this temperature effect is not clearly seen with SCRs due to the very low CI-OXPHOS rates used for their calculations resulting in disproportionately high SCRs (Figure 3C). It has been demonstrated that the initiation of flight in Colorado potato beetles requires relatively high temperatures, with a greater number of flights occurring between 20 and 30°C [reviewed in (Boiteau, 2001)]. Thus, our results indicate that at optimal flight temperature (between 18 and 30°C) proline is the preferred substrates in these beetles. Oxidation of proline is however sensitive to higher temperatures as OCRs declined sharply above 30°C.

In beetles, CI + ProDH-OXPHOS exhibited an unstable OCR at 30 and 42°C (hatched bars in Figure 1C). Jørgensen et al. (2021) also observed a similar instability of OCR but with CI-OXPHOS and suggested that this represents a transition state from normal to reduced CI-OXPHOS due to elevated temperature. Interestingly, this phenomenon did not happen at 36°C which might indicate an individual-specific thermal sensitivity that supports the transition hypothesis. These similarities between CI-OXPHOS for some *Drosophila* species and CI + ProDH-OXPHOS in potato beetles might indicate that proline is used for its anaplerotic role as electron donor for CI instead of for fuelling ProDH in this species (Bursell, 1963; Sacktor and Childress, 1967; Soares et al., 2015; McDonald et al., 2018; Stec et al., 2021), although we cannot directly confirm which pathway is activated for this substrate. Nevertheless, our results confirm that proline is an obligate mitochondrial substrate for flight in beetles at average temperatures, which can be an adaptation to nutritional resources of these insects that feed on potato plants. This reliance may also represent a promising strategy to control their flight dispersion through insecticidal double-stranded RNAs targeting the proline pathway.

### 4.3 Complex II and mtG3PDH Compensate for Decreased Complex I-Oxidative Phosphorylation in Honeybees and Fruit Flies

Complex II and mtG3PDH sustained moderately high to very high OCRs in fruit flies and honeybees respectively, particularly at high temperatures (Figure 1). Succinate contribution to OCR is higher in honeybees than in fruit flies at high temperatures (>30°C), as seen with the SCRs (Figure 3). In both species, these elevated SCRs are in part due to a ‘masking’ effect resulting from the use of low CI + ProDH-OXPHOS at high temperatures for their calculation, as previously shown (Jørgensen et al., 2021). Nevertheless, succinate is also clearly an important substrate for honeybees and fruit flies from 36°C and upwards. Interestingly, it has been observed that in honeybees, succinate does not stimulate oxygen consumption when measured at 24°C in isolated mitochondria (Syromyatnikov et al., 2019), possibly due to a limitation of succinate transport inside the mitochondrial matrix in insect flight muscle (Sacktor, 1955). However, Hedges et al. (2019) demonstrated succinate oxidation at 35°C in permeabilized thoraces of honeybees (albeit to a smaller extent than the present study) and wasps. Thus, succinate seems to be a specific substrate used at high temperatures in some insects capable of flight, and a possible explanation for the discrepancies observed for honeybees with other studies might reflect differences in specific environmental adaptations and/or genetic divergences. For example, flight metabolic rates of different subspecies of *A. mellifera* could vary as much as 20% (Harrison et al., 1996). In addition to its contribution at high temperatures, the higher SCR of succinate at 6°C in comparison to 12–30°C observed in fruit flies might reflect a role in short term cold tolerance. Accordingly, it has been shown that succinate compensated for a CI-OXPHOS decrease in the planarian *Dugesia tigrina* when measured at 10°C compared to 30°C (Scott et al., 2019). Moreover, succinate is a metabolite that can accumulate during anaerobic metabolism in several invertebrate species including some insect developmental stages, although anaerobic metabolism is not intensively studied in insects (Müller et al., 2012; Verberk et al., 2013; Harrison et al., 2018). However, considering that honeybees are experts at long-distance flight which is dependent of aerobic metabolism (Suarez et al., 1996), this increased oxidation of succinate is unlikely the result of a switch to anaerobic metabolism (at least for temperatures ∼35°C).

G3P is an important oxidative substrate at both low (6°C) and high (≥36°C) temperatures in honeybees and fruit flies, and at high (≥36°C) temperatures for beetles (Figures 1, 3). This is consistent with other studies in fruit flies showing high G3P contribution to mitochondrial respiration (Pichaud et al., 2010; Pichaud et al., 2011; Pichaud et al., 2013; Cormier et al., 2021; Jørgensen et al., 2021). Hedges et al. (2019) observed a mtG3PDH contribution to OCR equivalent to that of CI in *A. mellifera* but a higher one in the wasp *Vespula germanica* when measured at 35°C. Thus, our results confirm the metabolic specificity of insect flight muscles that have a high dependence on the mitochondrial/cytosolic G3PDH shuttle in comparison to other animals (Mráček et al., 2013). Although mtG3PDH theoretically has a lower energetic efficiency than CI in terms of ATP production (McDonald et al., 2018), the enzymatic activity of the cytosolic G3PDH is higher than all the other glycolytic enzymes in several orchid bees (Suarez et al., 2005). Honeybees are also known to perform thermogenesis for pre-flight warm-up and during winter (Stabentheiner et al., 2003), and it has been suggested that G3P is used in bumblebees for pre-flight thermogenesis at low temperatures (Masson et al., 2017). However, the small size of fruit flies precludes this process, which results in the inability to fly at temperatures below ∼14°C (Dudley, 2002; Frazier et al., 2008). Thus, our results on G3P SCRs at low temperatures might suggest a conserved adaptive mechanism between these two species and a role of the mtG3PDH in cold adaptation. At high temperatures, the three species also have higher G3P oxidation compared to the mid-range temperatures tested, albeit it is far more pronounced in fruit flies. This could also likely reflect temperature adaptations of the G3P shuttle to keep pace with the different metabolic demand at high temperatures in flying insects. Phylogenetic analysis of mtG3PDH including *Hymenoptera*, *Diptera*, and *Coleoptera* as well as other orders of flying insects in different thermal habitats could shed light on this potential adaptation. Our results are also consistent with the fact that mtG3PDH is most active in most highly energy demanding tissue such as the flight muscle (Mráček et al., 2013). We presume that this high level of G3P oxidation in honeybees and fruit flies is related to their greater flight activity in comparison to potato beetles. However, whether this allows to generate more ATP is to be confirmed.

### 4.4 Mitochondria and Organismal Thermal Limits

CIV maximal consumption rate steadily increased with temperature and plateaued in all three species from 24–30°C and upwards (Figure 4). The only exception to this pattern is fruit flies in which oxygen consumption decreased sharply at 45°C (Figure 4). Jørgensen et al. (2019) determined that the critical thermal maximum (CT_max_) of *D. melanogaster* is 38.3°C when acclimated to 19°C. However, we expect this CT_max_ to be a bit higher in our flies acclimated to 24°C. On the other hand, it has been shown that the CT_max_ of field-acclimated *A. mellifera carnica* is ∼50°C (Kovac et al., 2014), which is beyond the range of assay temperatures used in our study and thus, we could not measure at what temperature mitochondrial respiration was no longer sustained in this species. It is important to note here that honeybees were kept in laboratory control conditions (and not as a colony) at 24°C for the sake of comparing the different species at one common rearing temperature, which is lower than their optimal temperature. Although we did not find studies specifically measuring CT_max_ in adult potato beetles, some evidence suggest that it could be higher than 43–46°C (Chen et al., 2014; Chen et al., 2016). The fact that the thermal range for mitochondrial respiration failure in the fruit fly was close to its CT_max_ and that it was not detected in honeybees nor in beetles with higher CT_max_ might indicate a link between these two parameters although the causative relation is not known. Moreover, the decreased CI-OXPHOS and mitochondrial substrate switch occurring at high temperatures in honeybees are unlikely related to their thermal limits, but rather to a specific adaptive endothermic capacity allowing them to sustain energetic metabolism under thermal constrains.

### 4.5 Flexibility and Capacity of Energetic Metabolism in the Three Insect Species

When normalised to CIV maximal oxygen consumption rate, our results show that mitochondrial oxygen consumption differs between honeybees, fruit flies and beetles (Figure 5). This difference is mainly driven by the differential involvement of the mitochondrial complexes fuelling electrons to the ETS. Specifically, fruit flies’ mitochondria are the most active during CI-OXPHOS and CI + ProDH + CII + mtG3PDH-OXPHOS. On the other hand, beetles’ mitochondria are most active during CI + ProDH-OXPHOS. This is surprising as honeybees are considered one of the most active flying insects since they have to forage over long distances and carry heavy loads that requires an increase of 40% of their metabolic rate and a three-fold increase of their power output (Wolf et al., 1989; Roberts and Elekonich, 2005). These results shed light on the high efficiency of fruit flies’ mitochondria especially in comparison to honeybees and the difference in strategies used by these different insects: honeybees have moderately high normalized mitochondrial oxygen consumption rates that are able to keep up with high energetic demands at high temperatures; fruit flies exhibit high mitochondrial metabolism which is less resilient to high temperatures; and potato beetle have less active flight muscles but are rather resilient to high temperatures.

It is important to stress that our study assessed mitochondrial responses to temperature in a summer (active) phenotype, and during conditions not necessarily optimal for each species, but to better characterize their phenotypic plasticity and capacity in the context of rapid thermal hardening. Therefore, low temperatures used in this study cannot be used to extrapolate the mitochondrial responses to thermal fluctuations observed in winter. For example, potato beetles degrade their mitochondria and suppress their metabolism in winter while honeybees are active and keep the queen warm by forming a cluster with an inside temperature of 32–36°C (Stegwee et al., 1963; Stabentheiner et al., 2010).

In summary, our results demonstrate that mitochondrial respiration increased with increasing temperature and this respiration is sustained by proline in beetles (*L. decemlineata)*, mainly by G3P in fruit flies (*D. melanogaster)*, and by succinate and G3P in honeybees (*A. mellifera carnica)*. Moreover, OCRs when fuelled with all substrates were still intact at 45°C in honeybees and beetles, but drastically declined in fruit flies. This highlights the metabolic flexibility of insects’ mitochondria that can rely on the oxidation of a large set of substrates fuelling mitochondrial complexes with different thermal tolerances to withstand increasing temperatures. However, not all insects have this ability as demonstrated by our results. Therefore, honeybees seem to be better equipped to adapt to increasing temperature and meet their high energetic demands than fruit flies or potato beetles. It would be interesting to further our investigation by directly measuring ATP production at high temperatures to better understand the nature of the link between mitochondrial failure and thermal tolerance, given that it might be the strongest parameter linking both processes (Chung and Schulte, 2020).

## Data Availability

The raw data supporting the conclusions of this article will be made available by the authors, without undue reservation.

## References

[B1] Adzigbli L. Sokolov E. P. Ponsuksili S. Sokolova I. M. (2022). Tissue- and Substrate-Dependent Mitochondrial Responses to Acute Hypoxia–Reoxygenation Stress in a marine Bivalve (*Crassostrea G*). J. Exp. Biol. 225, jeb243304. 10.1242/JEB.243304 34904172

[B2] Bale J. S. Gerday C. Parker A. Marahiel M. A. Shanks I. A. Davies P. L. (2002). Insects and Low Temperatures: From Molecular Biology to Distributions and Abundance. Phil. Trans. R. Soc. Lond. B 357, 849–862. 10.1098/RSTB.2002.1074 12171648 PMC1693004

[B3] Beenakkers A. M. T. van der Horst D. J. van Marrewijk W. J. A. (1984). Insect Flight Muscle Metabolism. Insect Biochem. 14, 243–260. 10.1016/0020-1790(84)90057-X

[B4] Blier P. U. Lemieux H. Pichaud N. (2014). Holding Our Breath in Our Modern World: Will Mitochondria Keep the Pace with Climate Changes? Can. J. Zool. 92, 591–601. 10.1139/cjz-2013-0183

[B5] Boiteau G. Coleman W. (1996). Cold Tolerance in the Colorado Potato Beetle, Leptinotarsa Decemlineata (Say) (Coleoptera: Chrysomelidae). Can. Entomol. 128, 1087–1099. 10.4039/ENT1281087-6

[B6] Boiteau G. (2001). Recruitment by Flight and Walking in a One-Generation Colorado Potato Beetle (Coleoptera: Chrysomelidae) Environment. Environ. Entomol. 30, 306–317. 10.1603/0046-225X-30.2.306

[B7] Bundgaard A. James A. M. Joyce W. Murphy M. P. Fago A. (2018). Suppression of Reactive Oxygen Species Generation in Heart Mitochondria from Anoxic Turtles: The Role of Complex I S-Nitrosation. J. Exp. Biol. 221, jeb174391. 10.1242/JEB.174391 29496783 PMC5963835

[B8] Bursell E. (1963). Aspects of the Metabolism of Amino Acids in the Tsetse Fly, Glossina (Diptera). J. Insect Physiol. 9, 439–452. 10.1016/0022-1910(63)90054-4

[B9] Bursell E. (1981). “The Role of Proline in Energy Metabolism,” in Energy Metabolism in Insects (Boston, MA: Springer US), 135–154. 10.1007/978-1-4615-9221-1_5

[B10] Candy D. J. Becker A. Wegener G. (1997). Coordination and Integration of Metabolism in Insect Flight*. Comp. Biochem. Physiol. B: Biochem. Mol. Biol. 117, 497–512. 10.1016/S0305-0491(97)00212-5

[B11] Chamberlin M. E. (2004). Top-Down Control Analysis of the Effect of Temperature on Ectotherm Oxidative Phosphorylation. Am. J. Physiol. Regul. Integr. Comp. Physiol. 287, R794–R800. 10.1152/AJPREGU.00240.2004 15191905

[B12] Chen J. Alyokhin A. Mota-Sanchez D. Baker M. Whalon M. (2014). Variation in Fitness Among Geographically Isolated Colorado Potato Beetle (Coleoptera: Chrysomelidae) Populations. Ann. Entomol. Soc. Am. 107, 128–135. 10.1603/AN13018

[B13] Chen J. Kitazumi A. Alpuerto J. Alyokhin A. de Los Reyes B. (2016). Heat-Induced Mortality and Expression of Heat Shock Proteins in Colorado Potato Beetles Treated with Imidacloprid. Insect Sci. 23, 548–554. 10.1111/1744-7917.12194 25504556

[B14] Chung D. J. Sparagna G. C. Chicco A. J. Schulte P. M. (2018). Patterns of Mitochondrial Membrane Remodeling Parallel Functional Adaptations to thermal Stress. J. Exp. Biol. 221, jeb174458. 10.1242/JEB.174458/20683 29643174

[B15] Chung D. J. Bryant H. J. Schulte P. M. (2017). Thermal Acclimation and Subspecies-Specific Effects on Heart and Brain Mitochondrial Performance in a Eurythermal Teleost (*Fundulus H*). J. Exp. Biol. 220, 1459–1471. 10.1242/jeb.151217 28153980

[B16] Chung D. J. Schulte P. M. (2020). Mitochondria and the thermal Limits of Ectotherms. J. Exp. Biol. 223, jeb227801. 10.1242/jeb.227801 33109621 PMC10668358

[B17] Clark M. S. Worland M. R. (2008). How Insects Survive the Cold: Molecular Mechanisms-A Review. J. Comp. Physiol. B 178 (8), 917–933. 10.1007/S00360-008-0286-4 18584182

[B18] Colinet H. Hance T. Vernon P. Bouchereau A. Renault D. (2007). Does Fluctuating thermal Regime Trigger Free Amino Acid Production in the Parasitic Wasp Aphidius Colemani (Hymenoptera: Aphidiinae)? Comp. Biochem. Physiol. Part. A. Mol. Integr. Physiol. 147, 484–492. 10.1016/J.CBPA.2007.01.030 17347005

[B19] Cormier R. J. Strang R. Menail H. Touaibia M. Pichaud N. (2021). Systemic and Mitochondrial Effects of Metabolic Inflexibility Induced by High Fat Diet in *Drosophila M* . Insect Biochem. Mol. Biol. 133, 103556. 10.1016/j.ibmb.2021.103556 33626368

[B20] Cormier R. P. J. Champigny C. M. Simard C. J. St-Coeur P.-D. Pichaud N. (2019). Dynamic Mitochondrial Responses to a High-Fat Diet in *Drosophila M* . Sci. Rep. 9, 4531. 10.1038/s41598-018-36060-5 30872605 PMC6418259

[B21] Cox G. K. Gillis T. E. (2020). Surviving Anoxia: The Maintenance of Energy Production and Tissue Integrity during Anoxia and Reoxygenation. J. Exp. Biol. 223, jeb207613. 10.1242/jeb.207613 32651221

[B22] Davison T. F. Bowler K. (1971). Changes in the Functional Efficiency of Flight Muscle Sarcosomes during Heat Death of adultCalliphora Erythrocephala. J. Cel. Physiol. 78, 37–47. 10.1002/JCP.1040780107 4255709

[B23] Denlinger D. L. (2002). Regulation of Diapause. Annu. Rev. Entomol. 47, 93–122. 10.1146/ANNUREV.ENTO.47.091201.145137 11729070

[B24] Dudley R. (2002). The Biomechanics of Insect Flight. Princeton, NJ: Princeton University Press. 10.2307/J.CTV301G2X

[B25] Duman J. G. Wu D. W. Xu L. Tursman D. Olsen T. M. (1998). Adaptations of Insects to Subzero Temperatures. Q. Rev. Biol. 66, 387–410. 10.1086/417337

[B26] Ekström A. Sandblom E. Blier P. U. Cyr B. A. D. Brijs J. Pichaud N. (2017). Thermal Sensitivity and Phenotypic Plasticity of Cardiac Mitochondrial Metabolism in European Perch, *Perca F* . J. Exp. Biol. 220, 386–396. 10.1242/JEB.150698 27852753

[B27] El-Wadawi R. Bowler K. (1995). The Development of Thermotolerance Protects Blowfly Flight Muscle Mitochondrial Function from Heat Damage. J. Exp. Biol. 198, 2413–2421. 10.1242/JEB.198.11.2413 9320335

[B28] Fangue N. A. Richards J. G. Schulte P. M. (2009). Do Mitochondrial Properties Explain Intraspecific Variation in Thermal Tolerance? J. Exp. Biol. 212, 514–522. 10.1242/jeb.024034 19181899

[B29] Frazier M. R. Harrison J. F. Kirkton S. D. Roberts S. P. (2008). Cold Rearing Improves Cold-Flight Performance in Drosophila Viachanges in wing Morphology. J. Exp. Biol. 211, 2116–2122. 10.1242/JEB.019422 18552301

[B30] Gnaiger E. Lassnig B. Kuznetsov A. Rieger G. Margreiter R. (1998). Mitochondrial Oxygen Affinity, Respiratory Flux Control and Excess Capacity of Cytochrome C Oxidase. J. Exp. Biol. 201, 1129–1139. 10.1242/jeb.201.8.1129 9510525

[B31] González‐Tokman D. Córdoba‐Aguilar A. Dáttilo W. Lira‐Noriega A. Sánchez‐Guillén R. A. Villalobos F. (2020). Insect Responses to Heat: Physiological Mechanisms, Evolution and Ecological Implications in a Warming World. Biol. Rev. 95, 802–821. 10.1111/BRV.12588 32035015

[B32] Harada A. E. Healy T. M. Burton R. S. (2019). Variation in Thermal Tolerance and its Relationship to Mitochondrial Function across Populations of Tigriopus Californicus. Front. Physiol. 10, 213. 10.3389/FPHYS.2019.00213 30930787 PMC6429002

[B33] Harrison J. F. Fewell J. H. Roberts S. P. Hall H. G. (1996). Achievement of Thermal Stability by Varying Metabolic Heat Production in Flying Honeybees. Science 274, 88–90. 10.1126/SCIENCE.274.5284.88 8810252

[B34] Harrison J. F. Greenlee K. J. Verberk W. C. E. P. (2018). Functional Hypoxia in Insects: Definition, Assessment, and Consequences for Physiology, Ecology, and Evolution. Annu. Rev. Entomol. 63, 303–325. 10.1146/ANNUREV-ENTO-020117-043145 28992421

[B35] Harrison J. F. Roberts S. P. (2000). Flight Respiration and Energetics. Annu. Rev. Physiol. 62, 179–205. 10.1146/ANNUREV.PHYSIOL.62.1.179 10845089

[B36] Haunerland N. H. (1997). Transport and Utilization of Lipids in Insect Flight Muscles*. Comp. Biochem. Physiol. Part. B Mol. Integr. Physiol. 117, 475–482. 10.1016/S0305-0491(97)00185-5

[B37] Hedges C. P. Wilkinson R. T. Devaux J. B. L. Hickey A. J. R. (2019). Hymenoptera Flight Muscle Mitochondrial Function: Increasing Metabolic Power Increases Oxidative Stress. Comp. Biochem. Physiol. Part. A. Mol. Integr. Physiol. 230, 115–121. 10.1016/J.CBPA.2019.01.002 30677507

[B38] Hochachka P. W. Somero G. N. (2002). Biochemical Adaptation: Mechanism and Process in Physiological Evolution. New York: Oxford University Press. 10.1002/bmb.2002.494030030071

[B39] Hraoui G. Bettinazzi S. Gendron A. D. Boisclair D. Breton S. (2020). Mitochondrial Thermo-Sensitivity in Invasive and Native Freshwater Mussels. J. Exp. Biol. 223, jeb215921. 10.1242/jeb.215921 31915201

[B40] Hraoui G. Breton S. Miron G. Boudreau L. H. Hunter-Manseau F. Pichaud N. (2021). Mitochondrial Responses towards Intermittent Heat Shocks in the Eastern Oyster, *Crassostrea V* . J. Exp. Biol. 224, jeb242745. 10.1242/JEB.242745 34401903

[B41] Iftikar F. I. MacDonald J. Hickey A. J. R. (2010). Thermal Limits of Portunid Crab Heart Mitochondria: Could More Thermo-Stable Mitochondria Advantage Invasive Species? J. Exp. Mar. Biol. Ecol. 395, 232–239. 10.1016/J.JEMBE.2010.09.005

[B42] Iftikar F. I. MacDonald J. R. Baker D. W. Renshaw G. M. C. Hickey A. J. R. (2014). Could thermal Sensitivity of Mitochondria Determine Species Distribution in a Changing Climate? J. Exp. Biol. 217, 2348–2357. 10.1242/jeb.098798 25141346

[B43] Jena K. Kumar Kar P. Kausar Z. Babu C. S. (2013). Effects of Temperature on Modulation of Oxidative Stress and Antioxidant Defenses in Testes of Tropical Tasar Silkworm Antheraea Mylitta. J. Therm. Biol. 38, 199–204. 10.1016/J.JTHERBIO.2013.02.008

[B96] Jørgensen L. B. Malte H. Overgaard J. (2019). How to Assess Drosophila Heat Tolerance: Unifying Static and Dynamic Tolerance Assays to Predict Heat Distribution Limits. Funct. Ecol. 33 (4), 629–642.

[B44] Jørgensen L. B. Overgaard J. Hunter-Manseau F. Pichaud N. (2021). Dramatic Changes in Mitochondrial Substrate Use at Critically High Temperatures: A Comparative Study Using Drosophila. J. Exp. Biol. 224, jeb240960. 10.1242/jeb.240960 33563650

[B45] Kashmerry A. M. S. Bowler K. (1977). A Study of Recovery from Heat Injury in the Blowfly (*Calliphora E*) Using Split-Dose Experiments. J. Therm. Biol. 2, 183–184. 10.1016/0306-4565(77)90028-6

[B46] Koštál V. Korbelová J. Rozsypal J. Zahradníčková H. Cimlová J. Tomčala A. (2011). Long-Term Cold Acclimation Extends Survival Time at 0°C and Modifies the Metabolomic Profiles of the Larvae of the Fruit Fly *Drosophila M* . PLoS One 6, e25025. 10.1371/JOURNAL.PONE.0025025 21957472 PMC3177886

[B47] Koštál V. Šimek P. Zahradníčková H. Cimlová J. Štětina T. (2012). Conversion of the Chill Susceptible Fruit Fly Larva (*Drosophila M*) to a Freeze Tolerant Organism. Proc. Natl. Acad. Sci. U.S.A. 109, 3270–3274. 10.1073/PNAS.1119986109 22331891 PMC3295325

[B48] Kovac H. Käfer H. Stabentheiner A. Costa C. (2014). Metabolism and Upper thermal Limits of *Apis M* Carnica and A. M. Ligustica. Apidologie 45, 664–677. 10.1007/S13592-014-0284-3/TABLES/1 25378763 PMC4218932

[B49] Kuznetsov A. V. Veksler V. Gellerich F. N. Saks V. Margreiter R. Kunz W. S. (2008). Analysis of Mitochondrial Function *In Situ* in Permeabilized Muscle Fibers, Tissues and Cells. Nat. Protoc. 3, 965–976. 10.1038/nprot.2008.61 18536644

[B50] Lalouette L. Koštál V. Colinet H. Gagneul D. Renault D. (2007). Cold Exposure and Associated Metabolic Changes in Adult Tropical Beetles Exposed to Fluctuating thermal Regimes. FEBS J. 274, 1759–1767. 10.1111/J.1742-4658.2007.05723.X 17331186

[B51] Lee R. E. Chen C.-P. Denlinger D. L. (1987). A Rapid Cold-Hardening Process in Insects. Science 238, 1415–1417. 10.1126/SCIENCE.238.4832.1415 17800568

[B52] Loli D. Bicudo J. E. P. W. (2005). Control and Regulatory Mechanisms Associated with Thermogenesis in Flying Insects and Birds. Biosci. Rep. 25, 149–180. 10.1007/S10540-005-2883-8 16283551

[B53] Martin K. E. Currie S. Pichaud N. (2021). Mitochondrial Physiology and Responses to Elevated Hydrogen Sulphide in Two Isogenic Lineages of an Amphibious Mangrove Fish. J. Exp. Biol. 224, jeb241216. 10.1242/JEB.241216/237801 33688059

[B54] Martinez E. Hendricks E. Menze M. A. Torres J. J. (2016). Physiological Performance of Warm-Adapted marine Ectotherms: Thermal Limits of Mitochondrial Energy Transduction Efficiency. Comp. Biochem. Physiol. Part. A. Mol. Integr. Physiol. 191, 216–225. 10.1016/J.CBPA.2015.08.008 26297983

[B55] Masson S. W. C. Hedges C. P. Devaux J. B. L. James C. S. Hickey A. J. R. (2017). Mitochondrial Glycerol 3-Phosphate Facilitates Bumblebee Pre-Flight Thermogenesis. Sci. Rep. 7, 1–7. 10.1038/s41598-017-13454-5 29026172 PMC5638826

[B56] McDonald A. E. Pichaud N. Darveau C.-A. (2018). "Alternative" Fuels Contributing to Mitochondrial Electron Transport: Importance of Non-Classical Pathways in the Diversity of Animal Metabolism. Comp. Biochem. Physiol. Part. B Mol. Integr. Physiol. 224, 185–194. 10.1016/J.CBPB.2017.11.006 29155008

[B57] Michaud M. R. Denlinger D. L. (2007). Shifts in the Carbohydrate, Polyol, and Amino Acid Pools during Rapid Cold-Hardening and Diapause-Associated Cold-Hardening in Flesh Flies (Sarcophaga Crassipalpis): A Metabolomic Comparison. J. Comp. Physiol. B 177, 753–763. 10.1007/S00360-007-0172-5/FIGURES/6 17576567

[B58] Micheu S. Crailsheim K. Leonhard B. (2000). Importance of Proline and Other Amino Acids during Honeybee Flight. Amino Acids 18, 157–175. 10.1007/S007260050014 10817408

[B59] Moyes C. D. Suarez R. K. Hochachka P. W. Ballantyne J. S. (1990). A Comparison of Fuel Preferences of Mitochondria from Vertebrates and Invertebrates. Can. J. Zool. 68, 1337–1349. 10.1139/Z90-201

[B60] Mráček T. Drahota Z. Houštěk J. (2013). The Function and the Role of the Mitochondrial Glycerol-3-Phosphate Dehydrogenase in Mammalian Tissues. Biochim. Biophys. Acta (Bba) - Bioenerg. 1827, 401–410. 10.1016/j.bbabio.2012.11.014 23220394

[B61] Müller M. Mentel M. van Hellemond J. J. Henze K. Woehle C. Gould S. B. (2012). Biochemistry and Evolution of Anaerobic Energy Metabolism in Eukaryotes. Microbiol. Mol. Biol. Rev. 76, 444–495. 10.1128/MMBR.05024-11 22688819 PMC3372258

[B62] Overgaard J. Macmillan H. A. (2017). The Integrative Physiology of Insect Chill Tolerance. Annu. Rev. Physiol. 79, 187–208. 10.1146/ANNUREV-PHYSIOL-022516-034142 27860831

[B63] Overgaard J. Malmendal A. Sørensen J. G. Bundy J. G. Loeschcke V. Nielsen N. C. (2007). Metabolomic Profiling of Rapid Cold Hardening and Cold Shock in *Drosophila M* . J. Insect Physiol. 53, 1218–1232. 10.1016/J.JINSPHYS.2007.06.012 17662301

[B64] Pesta D. Gnaiger E. (2012). High-Resolution Respirometry: OXPHOS Protocols for Human Cells and Permeabilized Fibers from Small Biopsies of Human Muscle. Methods Mol. Biol. 810, 25–58. 10.1007/978-1-61779-382-0_3 22057559

[B65] Pichaud N. Ballard J. W. O. Tanguay R. M. Blier P. U. (2013). Mitochondrial Haplotype Divergences Affect Specific Temperature Sensitivity of Mitochondrial Respiration. J. Bioenerg. Biomembr 45, 25–35. 10.1007/s10863-012-9473-9 23054075

[B66] Pichaud N. Ballard J. W. O. Tanguay R. M. Blier P. U. (2012). Naturally Occurring Mitochondrial Dna Haplotypes Exhibit Metabolic Differences: Insight into Functional Properties of Mitochondria. Evolution (N Y) 66, 3189–3197. 10.1111/j.1558-5646.2012.01683.x 23025608

[B67] Pichaud N. Ballard J. W. O. Tanguay R. M. Blier P. U. (2011). Thermal Sensitivity of Mitochondrial Functions in Permeabilized Muscle Fibers from Two Populations of *Drosophila S* with Divergent Mitotypes. Am. J. Physiol. Regul. Integr. Comp. Physiol. 301, R48–R59. 10.1152/ajpregu.00542.2010 21451139

[B68] Pichaud N. Chatelain E. H. Ballard J. W. O. Tanguay R. Morrow G. Blier P. U. (2010). Thermal Sensitivity of Mitochondrial Metabolism in Two Distinct Mitotypes of *Drosophila S*: Evaluation of Mitochondrial Plasticity. J. Exp. Biol. 213, 1665–1675. 10.1242/jeb.040261 20435817

[B69] Pichaud N. Ekström A. Breton S. Sundström F. Rowinski P. Blier P. U. (2019). Cardiac Mitochondrial Plasticity and thermal Sensitivity in a Fish Inhabiting an Artificially Heated Ecosystem. Sci. Rep. 9, 17832. 10.1038/s41598-019-54165-3 31780821 PMC6883045

[B70] Roberts S. P. Elekonich M. M. (2005). Muscle Biochemistry and the Ontogeny of Flight Capacity during Behavioral Development in the Honey Bee, *Apis m* . J. Exp. Biol. 208, 4193–4198. 10.1242/JEB.01862 16272241

[B71] Sacktor B. (1955). Cell Structure and the Metabolism of Insect Flight Muscle. J. Biophys. Biochem. Cytol. 1, 29–46. 10.1083/jcb.1.1.29 14381426 PMC2223591

[B72] Sacktor B. Childress C. C. (1967). Metabolism of Proline in Insect Flight Muscle and its Significance in Stimulating the Oxidation of Pyruvate. Arch. Biochem. Biophys. 120, 583–588. 10.1016/0003-9861(67)90522-X

[B73] Sacktor B. Cochran D. G. (1958). The Respiratory Metabolism of Insect Flight Muscle. I. Manometric Studies of Oxidation and Concomitant Phosphorylation with Sarcosomes. Arch. Biochem. Biophys. 74, 266–276. 10.1016/0003-9861(58)90219-4 13522243

[B74] Schulte P. M. (2015). The Effects of Temperature on Aerobic Metabolism: towards a Mechanistic Understanding of the Responses of Ectotherms to a Changing Environment. J. Exp. Biol. 218, 1856–1866. 10.1242/jeb.118851 26085663

[B75] Scott K. Y. Matthew R. Woolcock J. Silva M. Lemieux H. (2019). Adjustments in the Control of Mitochondrial Respiratory Capacity to Tolerate Temperature Fluctuations. J. Exp. Biol. 222, jeb207951. 10.1242/JEB.207951 31439652

[B76] Simard C. J. Pelletier G. Boudreau L. H. Hebert-Chatelain E. Pichaud N. (2018). Measurement of Mitochondrial Oxygen Consumption in Permeabilized Fibers of Drosophila Using Minimal Amounts of Tissue. JoVE 134, e57376. 10.3791/57376 PMC5933415 29683457

[B77] Sinclair B. J. Marshall K. E. (2018). The Many Roles of Fats in Overwintering Insects. J. Exp. Biol. 221, jeb161836. 10.1242/JEB.161836/33964 29514877

[B78] Soares J. B. R. C. Gaviraghi A. Oliveira M. F. Veuthey J. Zamboni N. Westermann B. (2015). Mitochondrial Physiology in the Major Arbovirus Vector *Aedes A*: Substrate Preferences and Sexual Differences Define Respiratory Capacity and Superoxide Production. PLoS One 10, e0120600. 10.1371/journal.pone.0120600 25803027 PMC4372595

[B79] Sokolova I. (2018). Mitochondrial Adaptations to Variable Environments and Their Role in Animals' Stress Tolerance. Integr. Comp. Biol. 58, 519–531. 10.1093/ICB/ICY017 29701785

[B80] Stabentheiner A. Kovac H. Brodschneider R. (2010). Honeybee Colony Thermoregulation - Regulatory Mechanisms and Contribution of Individuals in Dependence on Age, Location and Thermal Stress. PLoS One 5, e8967. 10.1371/JOURNAL.PONE.0008967 20126462 PMC2813292

[B81] Stabentheiner A. Kovac H. Hetz S. K. Käfer H. Stabentheiner G. (2012). Assessing Honeybee and Wasp Thermoregulation and Energetics-New Insights by Combination of Flow-Through Respirometry with Infrared Thermography. Thermochim. Acta 534, 77–86. 10.1016/J.TCA.2012.02.006 22723718 PMC3378207

[B82] Stabentheiner A. Pressl H. Papst T. Hrassnigg N. Crailsheim K. (2003). Endothermic Heat Production in Honeybee Winter Clusters. J. Exp. Biol. 206, 353–358. 10.1242/jeb.00082 12477904

[B83] Stec N. Saleem A. Darveau C.-A. (2021). Proline as a Sparker Metabolite of Oxidative Metabolism during the Flight of the Bumblebee, Bombus Impatiens. Metabolites 11, 511. 10.3390/METABO11080511 34436452 PMC8399816

[B84] Stegwee D. Kimmel E. C. de Boer J. A. Henstra S. (1963). Hormonal Control of Reversible Degeneration of Flight Muscle in the Colorado Potato Beetle, *Leptinotarsa D* Say (Coleoptera). J. Cel Biol. 19, 519–527. 10.1083/JCB.19.3.519 PMC2106340 19866636

[B85] Strachan L. A. Tarnowski-Garner H. E. Marshall K. E. Sinclair B. J. (2015). The Evolution of Cold Tolerance in Drosophila Larvae. Physiol. Biochem. Zool. 84, 43–53. 10.1086/657147 21050129

[B86] Suarez R. K. Darveau C.-A. Welch K. C. O'Brien D. M. Roubik D. W. Hochachka P. W. (2005). Energy Metabolism in Orchid Bee Flight Muscles: Carbohydrate Fuels All. J. Exp. Biol. 208, 3573–3579. 10.1242/jeb.01775 16155228

[B87] Suarez R. K. (2000). Energy Metabolism during Insect Flight: Biochemical Design and Physiological Performance. Physiol. Biochem. Zool. 73, 765–771. 10.1086/318112 11121349

[B88] Suarez R. K. Lighton J. R. Joos B. Roberts S. P. Harrison J. F. (1996). Energy Metabolism, Enzymatic Flux Capacities, and Metabolic Flux Rates in Flying Honeybees. Proc. Natl. Acad. Sci. U.S.A. 93, 12616–12620. 10.1073/pnas.93.22.12616 8901631 PMC38041

[B89] Suarez R. K. Staples J. F. Lighton J. R. Mathieu-Costello O. (2000). Mitochondrial Function in Flying Honeybees (*Apis M*): Respiratory Chain Enzymes and Electron Flow from Complex III to Oxygen. J. Exp. Biol. 203, 905–911. 10.1242/JEB.203.5.905 10667973

[B90] Syromyatnikov M. Y. Gureev A. P. Vitkalova I. Y. Starkov A. A. Popov V. N. (2019). Unique Features of Flight Muscles Mitochondria of Honey Bees (*Apis M* L.). Arch. Insect Biochem. Physiol. 102, e21595. 10.1002/ARCH.21595 31276240

[B91] Teets N. M. Denlinger D. L. (2013). Physiological Mechanisms of Seasonal and Rapid Cold-Hardening in Insects. Physiol. Entomol. 38, 105–116. 10.1111/PHEN.12019

[B92] Teulier L. Weber J.-M. Crevier J. Darveau C.-A. (2016). Proline as a Fuel for Insect Flight: Enhancing Carbohydrate Oxidation in Hymenopterans. Proc. R. Soc. B. 283, 20160333. 10.1098/rspb.2016.0333 PMC4947884 27412285

[B93] Verberk W. C. E. P. Sommer U. Davidson R. L. Viant M. R. (2013). Anaerobic Metabolism at Thermal Extremes: A Metabolomic Test of the Oxygen Limitation Hypothesis in an Aquatic Insect. Integr. Comp. Biol. 53, 609–619. 10.1093/ICB/ICT015 23604617 PMC3776598

[B94] Weeda E. de Kort C. A. D. Beenakkers A. M. T. (1980). Oxidation of Proline and Pyruvate by Flight Muscle Mitochondria of the Colorado Beetle, *Leptinotarsa D* Say. Insect Biochem. 10, 305–311. 10.1016/0020-1790(80)90025-6

[B95] Wolf T. J. Schmid-Hempel P. Ellington C. P. Stevenson R. D. (1989). Physiological Correlates of Foraging Efforts in Honey-Bees: Oxygen Consumption and Nectar Load. Funct. Ecol. 3, 417–424. 10.2307/2389615

