# Supplementary material for: Flexible Thermal Sensitivity of Mitochondrial Oxygen Consumption and Substrate Oxidation in Flying Insect Species
Source: Front Physiol. 2022 Apr 25;13:897174. doi: 10.3389/fphys.2022.897174 (PMC9081799; doi:10.3389/fphys.2022.897174)
Supplement: Supplementary file 1 [file DataSheet1.PDF]

Flexible thermal sensitivity of mitochondrial oxygen consumption and substrate oxidation in flying insect species

Hichem A. Menail[1,2], Simon B. Cormier[1,2], Mariem Ben Youssef[2], Lisa Bjerregaard Jørgensen[3], Jess L. Vickruck[4], Pier Jr Morin[2], Luc H. Boudreau[1,2], Nicolas Pichaud[1,2*]

[1]New Brunswick Centre for Precision Medicine, Moncton, NB, Canada, E1A 3E9

[2] Department of Chemistry and Biochemistry, Université de Moncton, Moncton, NB, Canada, E1 A 3E9.

[3]Zoophysiology, Department of Biology, Aarhus University, 8000 Aarhus C, Denmark

[4]Fredericton Research and Development Centre, Agriculture and Agri-Food Canada, 850 Lincoln Road, Fredericton, NB E3B 4Z7, Canada

* Corresponding author: nicolas.pichaud@umoncton.ca

Supplementary information

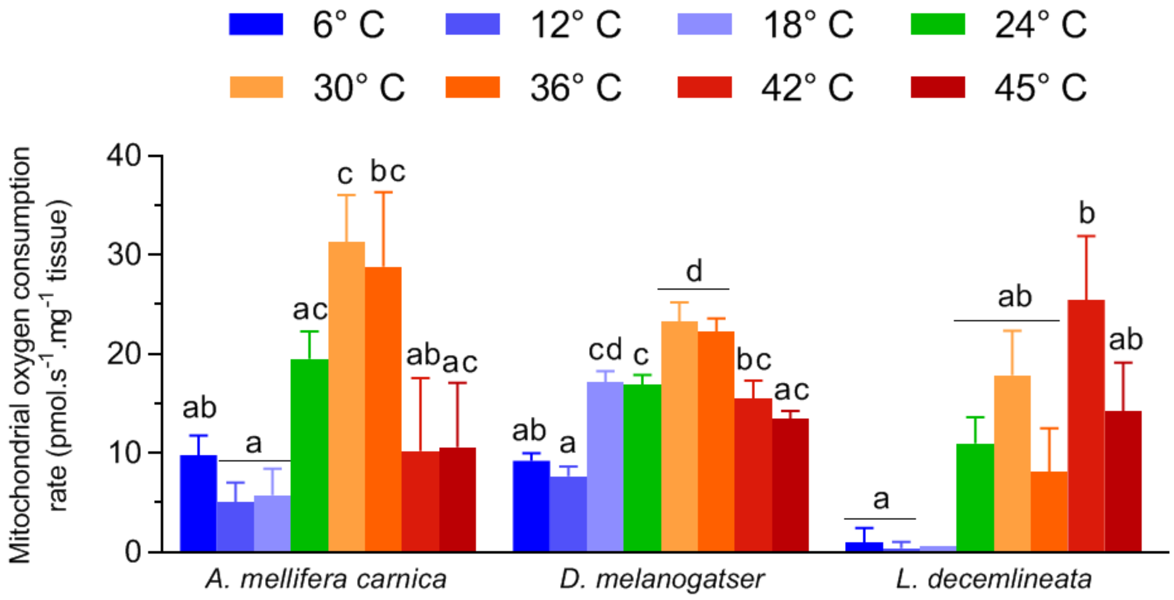

Figure S1. Thermal sensitivity of CI-LEAK measured from 6 to 45°C in permeabilized thoraces of *A. mellifera carnica,D. melanogaster*and *L. decemlineata*.CI-LEAK was measured with pyruvate+malate (+glutamate in honeybees),are reported as mean ± s.e.m., and were compared within each species between assay temperatures using a one-way ANOVA followed by a HSD (Honest Significant Difference) Tukey's test. Dissimilar letters indicate significant differences between CI-LEAK.

Table S1. Statistical analysis (F-test ANOVA/X^2Kruskal-Wallis) obtained for the different OCRs measured in the permeabilized thorax of the three insect species (*A. mellifera carnica*, *D. melanogaster* and *L. decemlineata*).

Respiration rates	*A. mellifera carnica*			*D. melanogaster*			*L. decemlineata*		
	**F**	**dF (residual)**	**p**	**F**	**dF (residual)**	**p**	**F**	**dF (residual)**	**p**
**CI-OXPHOS**	16.41	66	<0.0001	127.1	44	<0.0001	3.844	42	0.0026
**CI+ProDH-OXPHOS**	19.95	66	<0.0001	216.7	44	<0.0001	20.69	58	<0.0001
**CI+ProDH+CII-OXPHOS**	41.92	70	<0.0001	169.5	44	<0.0001	43.91†	60	<0.0001
**CI+ProDH+CII+mtG3PDH-OXPHOS**	30.70	70	<0.0001	83.26	44	<0.0001	17.63	60	<0.0001
**CIV**	21.49	70	<0.0001	119.1	44	<0.0001	44.06	60	<0.0001
**CI coupling efficiency**	3.77	58	0.002	83.05	39	<0.0001	1.67	34	0.1797

† X^2 value obtained after a Kruskal-Wallis test

Table S2. Statistical analysis (F-test ANOVA/ X^2 Kruskal-Wallis) calculated for the different SCRs with thedifferent OCRs measured in the permeabilized thorax of the three insect species (*A. mellifera carnica, D. melanogaster* and *L. decemlineata).*

Species	Substrate	F	dF (residual)	p
A. mellifera carnica	**Proline**	37.44†	69	<0.0001
	**Succinate**	17.15	63	<0.0001
	**G3P**	8.501	70	<0.0001
D. melanogaster	**Proline**	2.668	44	0.0216
	**Succinate**	101.2	44	<0.0001
	**G3P**	166.0	44	<0.0001
L. decemlineata	**Proline**	5.863	35	0.0001
	**Succinate**	26.67†	56	0.0004
	**G3P**	42.63†	58	<0.0001

† X^2 value obtained after a Kruskal-Wallis test

Table S3. Statistical analysis (F-test ANOVA/ X^2 Kruskal-Wallis) of relative OCRs calculated for between-species comparison by dividing each OCR by complex IV maximal oxygen consumption.

Temperature (° C)	Relative CI-OXPHOS			Relative CI+ProDH-OXPHOS			Relative CI+ProDH+CII-OXPHOS			Relative CI+ProDH+CII+mtG3PDH-OXPHOS		
	**F**	**dF (residual)**	**p**	**F**	**dF (residual)**	**p**	**F**	**dF (residual)**	**p**	**F**	**dF (residual)**	**p**
**6**	7.143	18	0.0052	11.01	21	0.0005	9.637	22	0.001	52.42	22	<0.0001
**12**	35.54	19	<0.0001	41.26	22	<0.0001	15.78	22	<0.0001	22.71	22	<0.0001
**18**	34.95	19	<0.0001	67.5	21	<0.0001	35.37	21	<0.0001	7.462	21	0.0036
**24**	58.61	19	<0.0001	60.94	22	<0.0001	47.52	22	<0.0001	26.49	22	<0.0001
**30**	47.25	20	<0.0001	19.93	19	<0.0001	10.92†		0.0042	67.33	21	<0.0001
**36**	5.338	22	0.0129	5.274	21	0.0139	3.013	25	0.0672	62.6	25	<0.0001
**42**	37.42	19	0.6928	2.307	21	0.1242	3.357	21	0.0543	186.1	21	<0.0001
**45**	7.017	17	0.006	7.971	19	0.0031	22.05	20	<0.0001	60.3	20	<0.0001

† X^2 value obtained after a Kruskal-Wallis test
